# Effective virus-neutralizing activities in antisera from the first wave of survivors of severe COVID-19

**DOI:** 10.1172/jci.insight.146267

**Published:** 2021-02-22

**Authors:** Yang Han, Peipei Liu, Yang Qiu, Jie Zhou, Ying Liu, Xujuan Hu, Qingyu Yang, Rui Huang, Xinyue Wen, Hao Song, Pengcheng Yu, Mengjie Yang, Jing Zhang, William J. Liu, Ke Peng, Guizhen Wu, Dingyu Zhang, Xi Zhou, Ying Wu

**Affiliations:** 1 Joint Laboratory of Infectious Diseases and Health, Wuhan Institute of Virology & Wuhan Jinyintan Hospital, Wuhan Jinyintan Hospital, Wuhan, Hubei, China.; 2 NHC Key Laboratory of Biosafety, National Institute for Viral Disease Control and Prevention, Chinese Center for Disease Control and Prevention, Beijing, China.; 3 State Key Laboratory of Virology, Wuhan Institute of Virology, Center for Biosafety Mega-Science, Chinese Academy of Sciences, Wuhan, Hubei, China.; 4 State Key Laboratory of Virology, Institute of Medical Virology and School of Basic Medical Sciences, Wuhan University, Wuhan, China.; 5 Research Network of Immunity and Health (RNIH), Beijing Institutes of Life Science, Chinese Academy of Sciences, Beijing, China.

**Keywords:** COVID-19, Adaptive immunity, Immunoglobulins

## Abstract

The coronavirus disease 19 (COVID-19) pandemic, caused by severe acute respiratory syndrome coronavirus 2 (SARS-CoV-2), has become the worst public health crisis in a century. However, knowledge about the dynamics of antibody responses in patients with COVID-19 is still poorly understood. In this study, we performed a serological study with serum specimens collected at the acute and the convalescent phases from 104 patients with severe COVID-19 who were part of the first wave of COVID-19 cases in Wuhan, China. Our findings revealed that neutralizing antibodies to SARS-CoV-2 are persistent for at least 6 months in patients with severe COVID-19, despite that IgG levels against the receptor binding domain (RBD) and nucleocapsid protein (N) IgG declined from the acute to the convalescent phase. Moreover, we demonstrate that the level of RBD-IgG is capable of correlating with SARS-CoV-2–neutralizing activities in COVID-19 serum. In summary, our findings identify the magnitude, functionality, and longevity of antibody responses in patients with COVID-19, which sheds light on the humoral immune response to COVID-19 and would be beneficial for developing vaccines.

## Graphical abstract

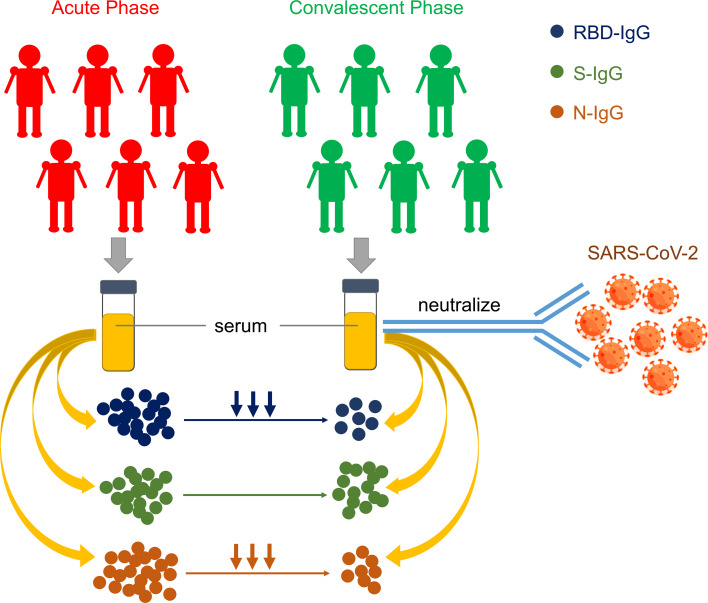

## Introduction

The Coronavirus Disease 19 (COVID-19) pandemic, caused by severe acute respiratory syndrome coronavirus 2 (SARS-CoV-2) (1–3), has become the worst public health crisis in a century. As of January 4, 2020, COVID-19 has infected nearly 90 million people and caused over 1.8 million deaths. SARS-CoV-2 is an enveloped, positive-strand RNA virus belonging to the β coronavirus genus and it is the seventh coronavirus that has infected humans so far (4, 5). In terms of clinical manifestations, most of the patients with COVID-19 have no symptoms or mild symptoms, such as cough, headache, and myalgia, but the disease course in some patients can progress rapidly to severe and even to critical illness (6).

Antibody response plays an important role in host resistance to viral diseases and reinfections and is tightly correlated with the convalescent processes of patients (7). Given the public health emergency and threat caused by the COVID-19 pandemic, it is critical to better understand the host antibody responses in patients with COVID-19, particularly those with severe symptoms. Thus far, dynamic changes of antibodies against SARS-CoV-2 in patients with COVID-19 have been mainly concentrated in patients who are asymptomatic or those with mild symptoms (8). However, in patients with severe COVID-19 symptoms, the effectiveness and durability of serum antibody protection after experiencing severe bodily damage requires more attention (9, 10). Additionally, this knowledge will be helpful for addressing the most urgent concerns, including reinfection, herd immunity, and vaccine efficacy.

The host-derived antibodies to SARS-CoV-2 have been found to target a variety of viral structural and nonstructural proteins (11, 12). Among all the viral antigens, 2 structural proteins — N protein and spike (S) protein — evoke the most common and robust antibody responses found in serum from patients with COVID-19 (13–15). N and S proteins are highly immunogenic antigens and frequently used in serological tests for SARS-CoV-2 (16–21). Furthermore, S protein is a large trimeric glycoprotein that contains the RBD (19, 22), which is required for SARS-CoV-2 to bind to the angiotensin-converting enzyme-2 receptor, thereby opening the door to entry into the target cells (23–25). A number of reports have shown that RBD is the target of the vast majority of neutralizing antibodies in convalescent serum (26–28). Moreover, a recent study identified that the correlation between anti-S and anti-N IgG was moderate, while the anti-RBD and anti-N IgG were better correlated (29).

Notably, the dynamic characteristics of the antibodies with neutralizing activity reflect the protective immune responses in patients with COVID-19 and the vaccinated population (11, 27). However, little is known about the magnitude, functionality, and longevity of neutralizing antibody responses in patients with COVID-19, especially in severe cases. Herein, we focused on 104 patients with severe COVID-19 who were among those of the first wave of COVID-19 in Wuhan and performed serological tests to measure the RBD-, N-, and S-IgG dynamic changes in serum approximately 6–7 months (median 195 days; IQR, 188–201 days) after disease onset. The correlation between RBD-IgG levels and neutralizing antibody titers in serum of patients with severe COVID-19 was also analyzed.

## Results

### Clinical characteristics of enrolled 104 patients with severe COVID-19.

We enrolled a cohort of 104 patients with COVID-19 who were previously admitted at Wuhan Jinyintan Hospital and diagnosed with severe conditions by the attending doctors according to the Chinese Health Commission (6th edition) (30). The disease onset time of these patients was between December 20, 2019 and January 27, 2020, the beginning of the first wave of the pandemic. The clinical and pathological characteristics of these patients are summarized in Supplemental Table 1 (supplemental material available online with this article; https://doi.org/10.1172/jci.insight.146267DS1). It is worth mentioning that all these patients were also enrolled in the clinical trial of lopinavir–ritonavir (31). Serum samples from these patients were collected at the acute phase and the convalescent phase, respectively. The median sample-collecting time of the acute phase for these patients was 23 days (IQR, 20–27 days) after the disease onset and that of the convalescent phase was 172 days (IQR, 167–176 days) after the acute phase sampling. In order to visualize the interval of each sampling point at the acute and the convalescent phase, sampling time-points for each patient were presented in the form of a stacked histogram (Supplemental Figure 1). Additionally, 31 healthy donors were also enrolled in the cohort as controls for comparison.

### Dynamic characteristics of antibodies in patients with severe COVID-19 at the acute and the convalescent phases.

**Figure 1:**
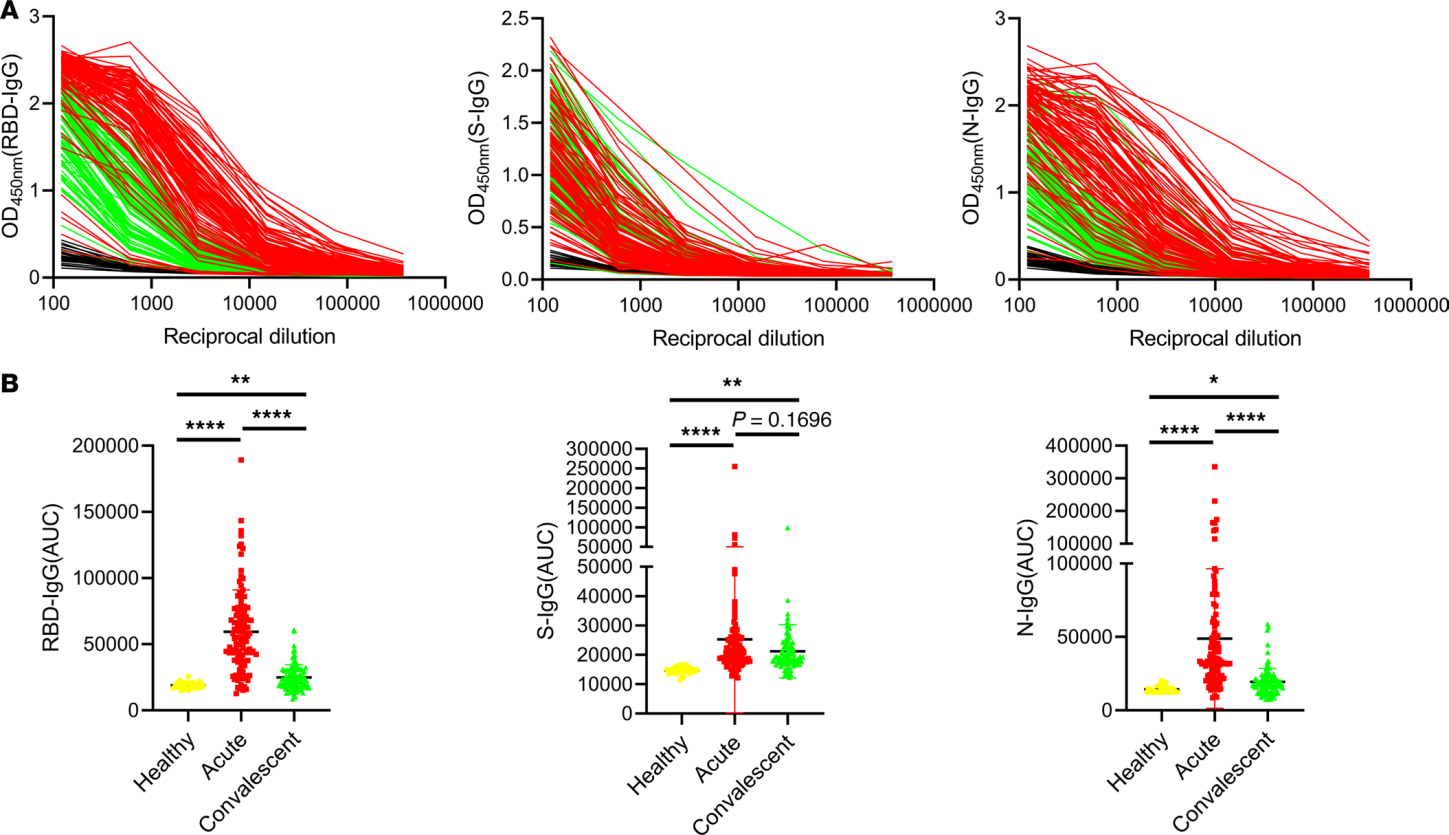
Calculation of antibodies in patients with severe COVID-19 at the acute and the convalescent phases. (**A**) ELISAs measuring antiserum reactivity to RBD, S, and N proteins were shown; Optical density units at 450 nm (OD, Y axis) and reciprocal plasma dilutions (X axis). The red lines represent the antibody dilution curves for the 104 patients with severe COVID-19 at the acute phase, the green lines are antibody dilution curves for these patients at the convalescent phase and the black curves indicate the antibody dilution in the 31 healthy subjects. (**B**) RBD-, S-, and N-IgG of patients with severe COVID-19 during acute and convalescent phases were shown and the results of healthy subjects by the same analysis were used as the control group. All kinds of IgGs were calculated according to the normalized AUC values in (**A**). The *P* value between any 2 groups was calculated by Student–Newman–Keuls multiple comparisons test, ANOVA, ****P < 0.0001, ***P < 0.001, **P < 0.01, *P < 0.05.

We examined the IgG levels against S, RBD, and N of SARS-CoV-2 by using ELISA assays, respectively. All the serum samples from 31 healthy donors and 104 patients with severe COVID-19 were serially diluted and the AUC of S-IgG, RBD-IgG, and N-IgG for each sample was measured based on the OD value at each dilution ratio, respectively. Of all the serum samples, one (Patient 15) was used as the internal reference in all tests for normalization of the AUC values in all further experiments (Figure 1A). As shown in Figure 1B, the averaged AUC values of RBD-IgG (24995 ± 9496) and N-IgG (19419 ± 9169) of patients with COVID-19 at the convalescent phase (green lines) were significantly lower than those at the acute phase (RBD-IgG: 59380 ± 31589; N-IgG: 48889 ± 47288; ****: *P* < 0.0001) (red lines), while the averaged AUC values of S-IgG at these 2 time-points showed no significant difference (acute phase: 25258 ± 24892, convalescent phase: 21209 ± 9069; *P* = 0.1696). In addition, the AUC values of RBD-, N-, and S-IgG from the convalescent or the acute serum were significantly higher than those from the healthy serum (*: *P* < 0.05; **: *P* < 0.01).

**Figure 2:**
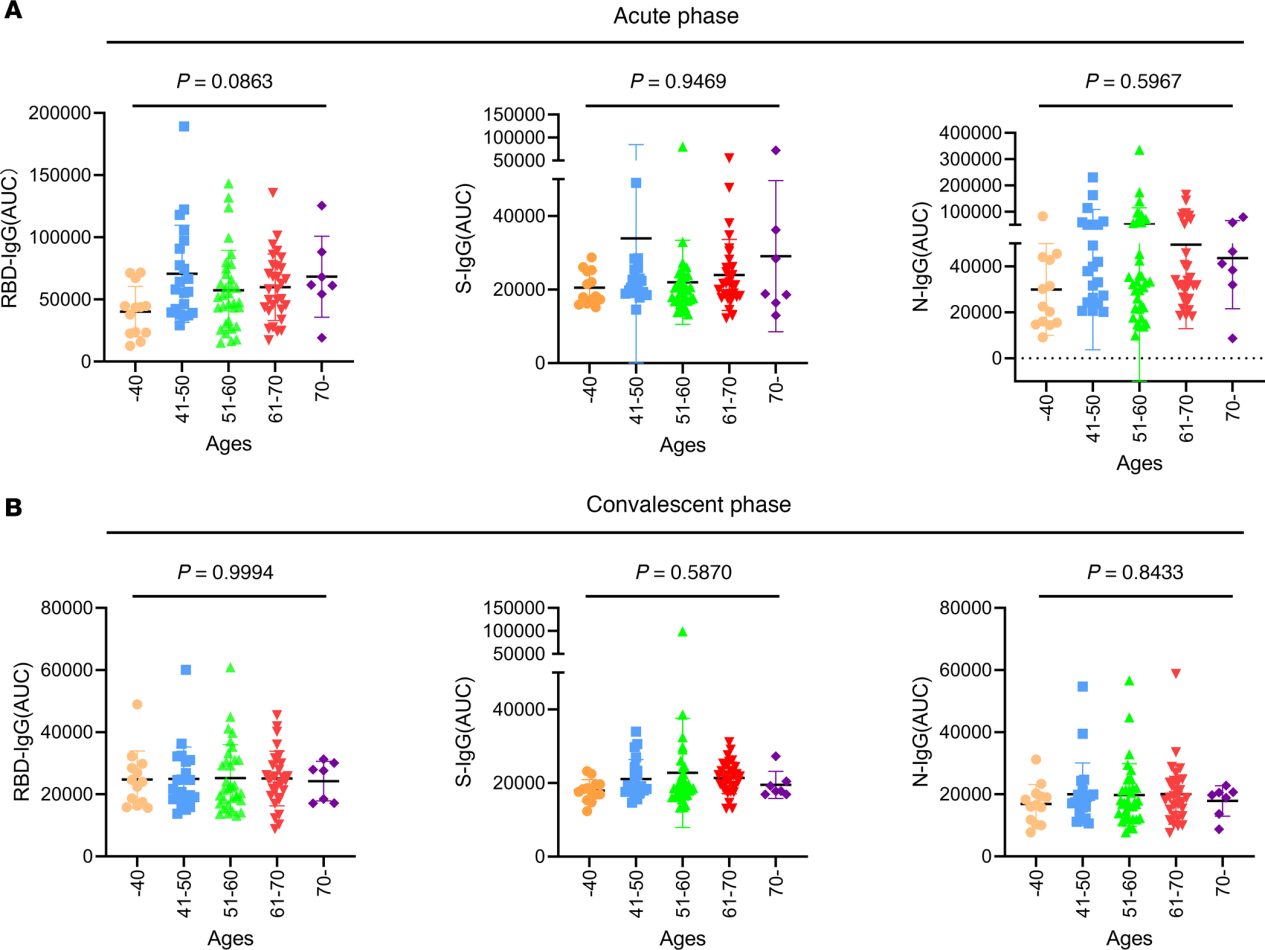
Antibody levels of different age groups in patients with severe COVID-19 at the acute and the convalescent phases. (**A** and **B**) The 104 patients with severe COVID-19 were divided into 5 groups by age (under 40 years, 41–50 years, 51–60 years, 61–70 years, and over 70 years). RBD-, S-, and N-IgG levels of each group at the acute (**A**) and the convalescent (**B**) phases were shown according to normalized AUC values. The *P* value among different groups was calculated by ANOVA analysis; *P* > 0.05, no significant difference.

**Figure 3:**
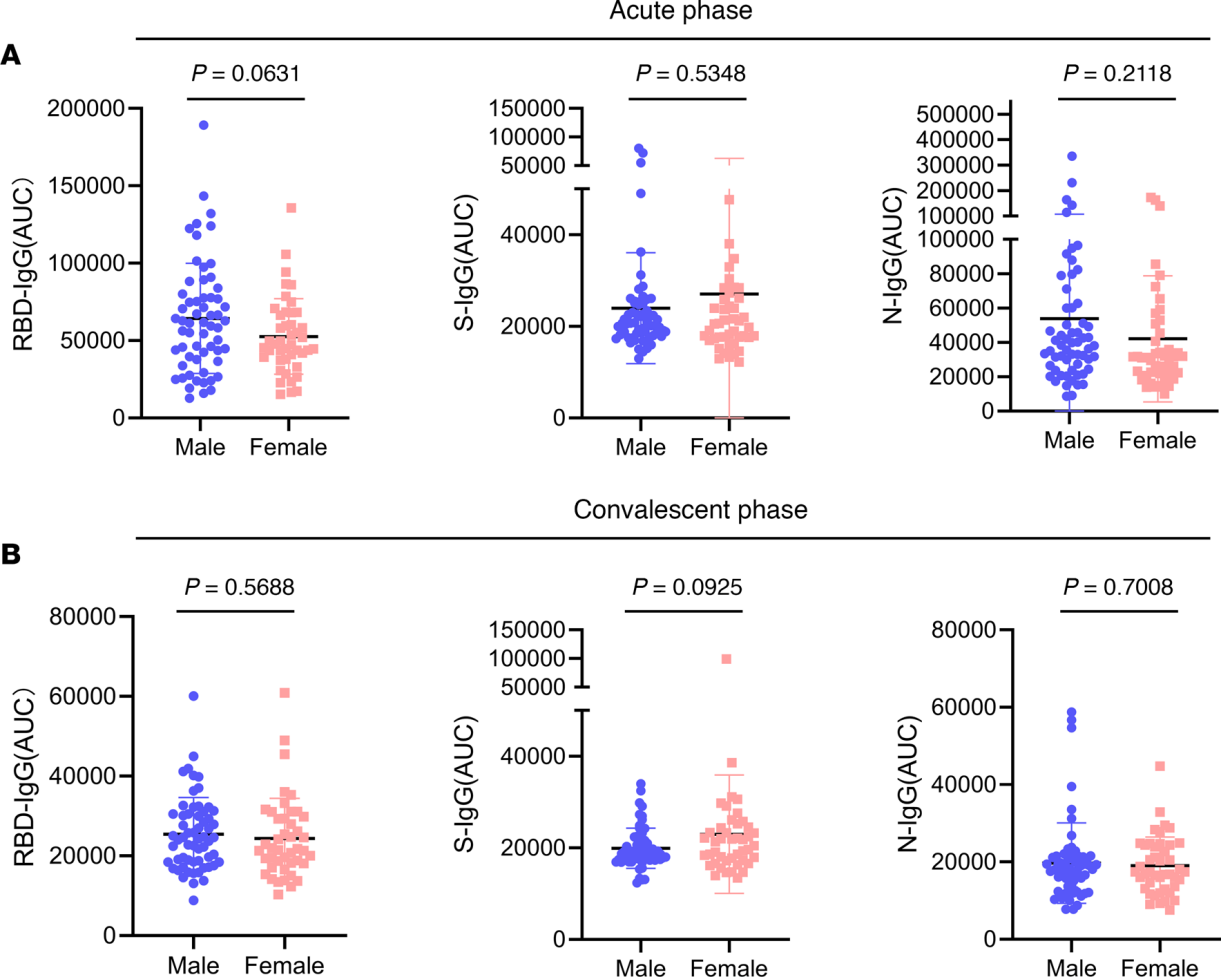
Antibody levels of different sex groups in patients with severe COVID-19 at the acute and the convalescent phases. (**A** and **B**) The 104 patients were divided into 2 groups by sex. Graphs showed RBD-, S-, and N-IgG levels of each group at the acute (**A**) and the convalescent (**B**) phase by normalized AUC values. The *P* value between any 2 groups was calculated by unpaired 2-tailed Student’s t test, *P* > 0.05, no significant difference.

Furthermore, we sought to explore whether the levels of the RBD-, S-, and N-IgG antibodies at the acute and the convalescent phase were related to age or sex. First, we divided the 104 samples into 5 groups based on patient age: under 40 years of age, 41–50 years of age, 51–60 years of age, 61–70 years of age, and over 70 years of age. Our results showed that there was no significant difference in RBD-, S- or N-IgG levels among the patients from different age groups either at the acute or the convalescent phase (*P* > 0.05, Figure 2, A and B). Subsequently, we divided these patients into male and female groups. Similarly, as a result, sex is not a decisive factor affecting the IgG levels at different phases (*P* > 0.05, Figure 3, A and B).

### Effective virus-neutralizing activities in the convalescent serum.

**Figure 4:**
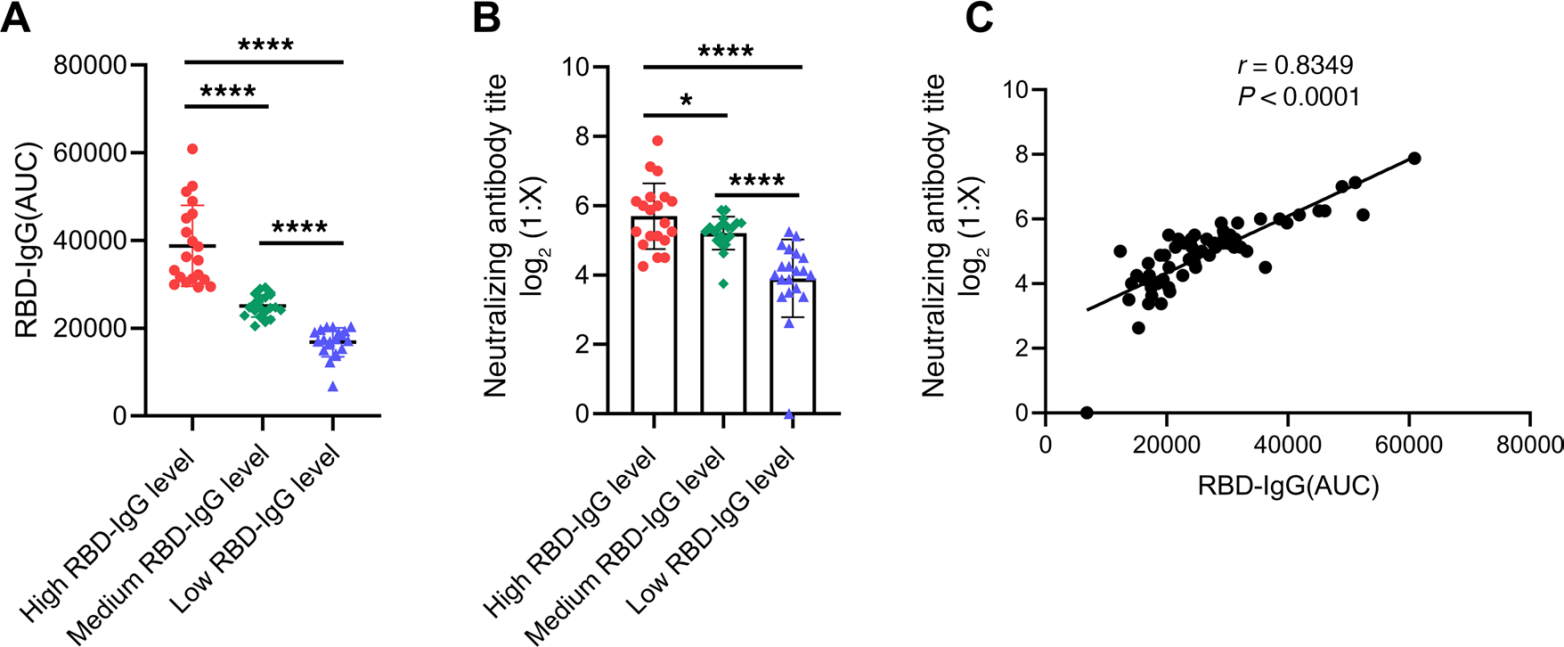
Analysis of correlation between RBD-IgG and neutralizing antibody titers. (**A**) According to the normalized AUC values, a total of 60 serum samples were divided into 3 groups based on the RBD-IgG levels of high, medium, and low. (**B**) Neutralizing antibody titers by using the authentic SARS-CoV-2 were detected for serum samples indicated by (**A**). The *P* value between any 2 groups in (**A**) and (**B**) was calculated by Student–Newman–Keuls multiple comparisons test, ANOVA, ****P < 0.0001, ***P < 0.001, **P < 0.01, *P < 0.05. (**C**) Correlation analysis between normalized AUC values of RBD-IgG and neutralizing antibody titers in the 60 serum samples from (**A** and **B**). There was a strong correlation between neutralizing antibody titer and RBD-IgG (*P* < 0.0001, *r* = 0.8349). Pearson correlation coefficient was used to determine the *r* value of the correlation between the 2 groups.

We sought to examine whether the convalescent serum still contains the neutralizing activity. To this end, we chose 60 samples from the 104 total samples according to high, medium, and low RBD-IgG AUC values and organized them into 3 groups of twenty samples each. Specifically, the AUC values of high, medium, and low RBD-IgG groups were ranked from the 6th to 20th (AUC: 60900 to 29472), the 43rd to 62nd (AUC: 28957 to 21443), and the 85th to 104th (AUC: 20497 to 6826) among the 104 patients, respectively. There were significant differences in RBD-IgG levels between any 2 groups (****: *P* < 0.0001, Figure 4A). Serum samples of high, medium, and low RBD-IgG level groups were then used to examine virus-neutralizing activity. A SARS-CoV-2 strain F13 (BetaCoV/Wuhan/IVDC-HB-envF13/2020) with very high titer and obvious evident cytopathic effect (CPE) when infected with Vero-E6 cells was used in the microneutralization assay. The overall titer of neutralizing activity of each sample was measured as the maximum reciprocal dilution at which the serum could inhibit 100 TCID_50_ SARS-CoV-2 completely. As a result, the titers of 96.7% (58/60) of these 60 samples were more than 8, indicating that most of the patients with severe COVID-19 still contain effective neutralizing activities even more than 6 months after disease onset.

Moreover, we revealed that the titers of virus-neutralizing antibodies of high, medium, and low RBD-IgG groups showed a downtrend with significant differences between them (*: *P* < 0. 05, ****: *P* < 0.0001, Figure 4B), consistent with the RBD-IgG AUC values. Meanwhile, in order to further determine the correlation between RBD-IgG levels and neutralizing antibody titers, correlation analysis was performed and the results showed that the neutralizing antibody titers were strongly correlated with RBD-IgG AUC values (*r* = 0.8349, *P* < 0.0001, Figure 4C).

### Correlation analysis between RBD-, S-, and N-IgG.

**Figure 5:**
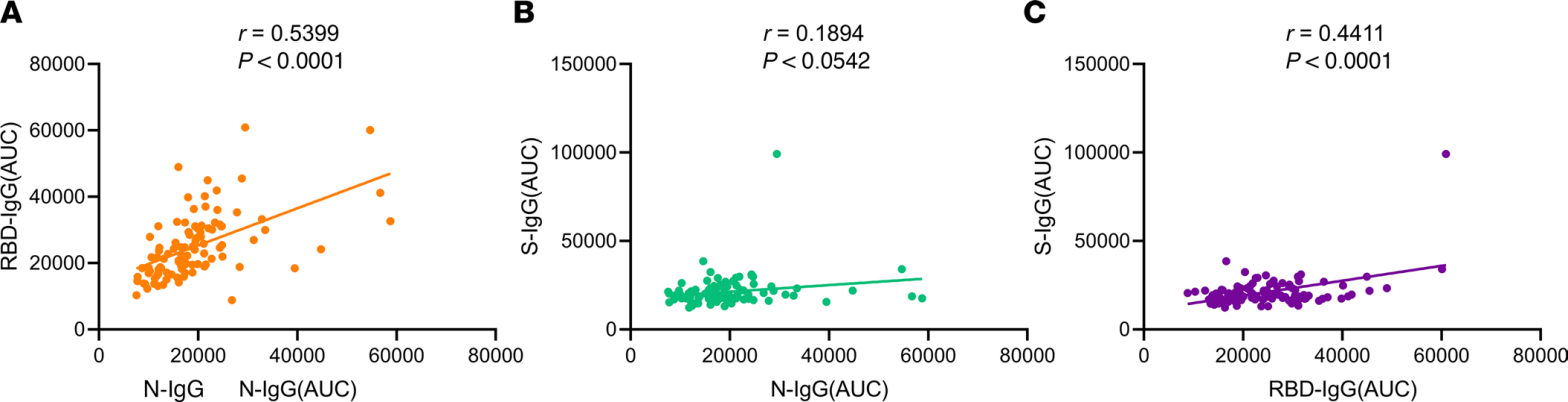
Correlation analysis among RBD-, S-, and N-IgG in patients with severe COVID-19 at the convalescent phase. According to normalized AUC values, correlation analysis of RBD-, S-, and N-IgG were performed. (**A**–**C**) RBD-IgG and N-IgG were moderately correlated (*r* = 0.5399, *P* < 0.0001; **A**), whereas RBD-IgG and S-IgG (*r* = 0.4411, *P* < 0.0001; **C**), and N-IgG and S-IgG (*r* = 0.1894, *P =* 0.0542; **B**) were not correlated. Pearson correlation coefficient was used to determine the *r* value of the correlation between any 2 groups.

We also performed the AUC value-based correlation analyses of RBD-, S-, and N-IgG at the convalescent phase and found that RBD-IgG and N-IgG were moderately correlated (*r* = 0.5399, *P* < 0.0001) (Figure 5), whereas RBD-IgG and S-IgG (*r* = 0.4411, *P* < 0.0001) and N-IgG and S-IgG (*r* = 0.1894, *P =* 0.0542) were not correlated. Given that the neutralizing antibody titers were strongly correlated with RBD-IgG levels, our findings indicated that the AUC values of RBD-IgG and N-IgG examined by ELISA were correlated with the titers of SARS-CoV-2–neutralizing antibodies.

### The decreased antibody levels in patients with severe COVID-19 at the convalescent phase.

**Figure 6:**
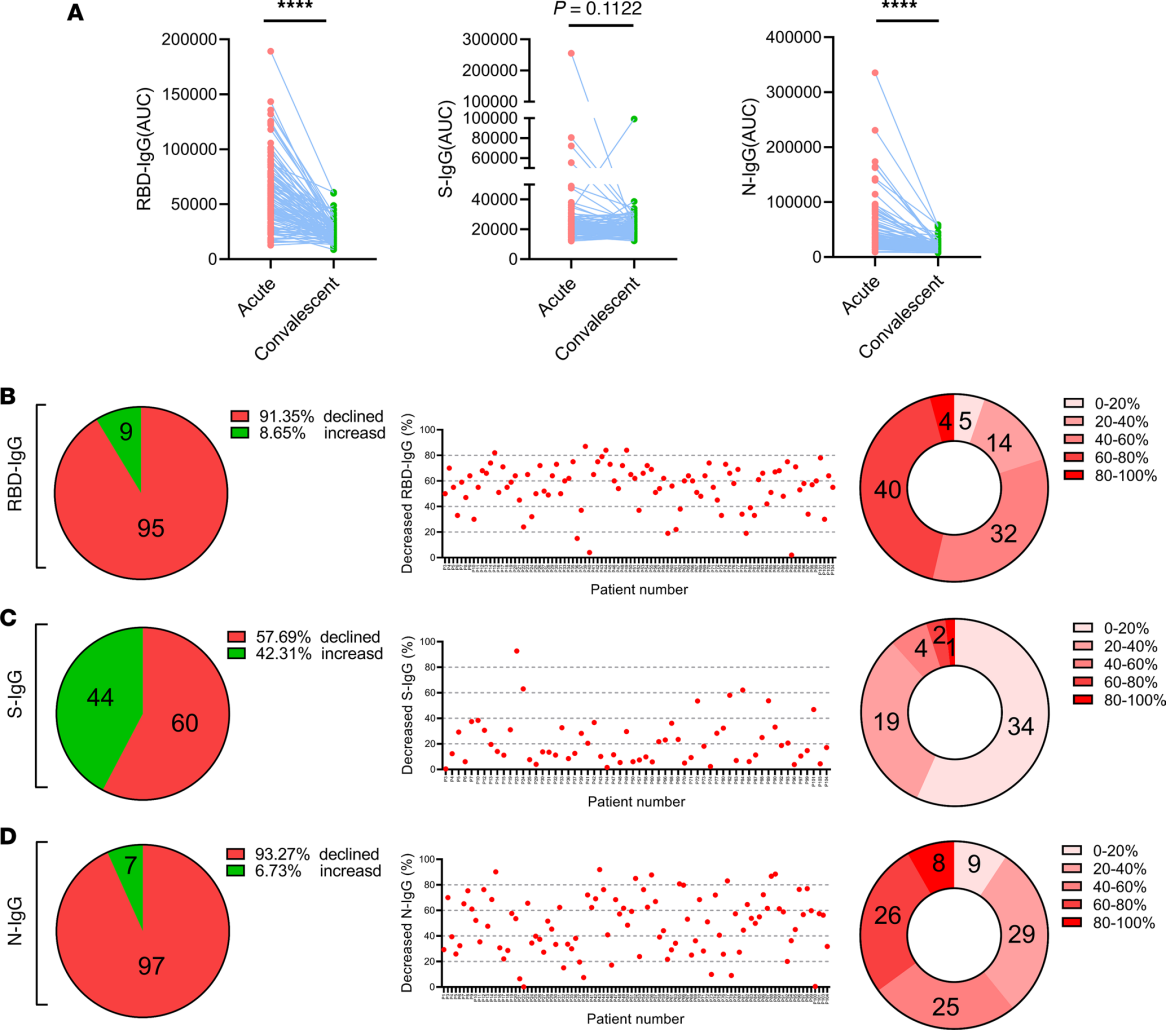
Analysis of the decreased tendency of IgG levels in patients with severe COVID-19. (**A**) Dynamic changes of normalized AUC values of RBD-, S-, and N-IgG levels during acute and convalescent phases. The *P* value was calculated by paired 2-tailed Student’s *t* tests. ****P < 0.0001, *P* > 0.05, no significant difference. (**B**–**D**) The left panel shows the number of patients with increased or decreased antibodies in the 104 patients, the middle panel shows the decreased percentage of antibodies in patients, the distribution of percentage of antibody reduction for these patients is shown in the right panel.

To further explore the antibody dynamic profiles of patients with severe COVID-19, we compared the AUC values of RBD-IgG, S-IgG, and N-IgG of 104 patients with COVID-19 at the acute and the convalescent phase and examined the details of the alterations of the antibody levels in each subject. As shown in Figure 6A, the levels of RBD- and N-IgG decreased significantly from the acute to the convalescent sampling points (paired 2-tailed Student’s t tests, ****: *P* < 0.0001, left and right panel), whereas the levels of S-IgG did not alter significantly at the different time-points (*P* = 0.1122, middle panel in Figure 6A). Moreover, the AUC values of RBD-IgG in 91.35% (95/104), S-IgG in 57.69% (60/104), and N-IgG in 93.27% (97/104) of the total patients were found to decline, respectively.

Additionally, we investigated the details of the percentages of decreased levels of RBD-, S-, and N-IgG in the 104 patients at the convalescent phase. The degree of the antibody that decreased in each patient was calculated by the following formula: the degree of decline in antibody (%) = [(AUC value of antibodies at the acute phase) – (AUC value of antibodies at the convalescent phase)] / (AUC value of antibodies at the acute phase). As a result, the median of the degree of reduction was 58.98% (IQR, 48.15%–68.25%) for RBD-IgG, 15.90% (IQR, 7.83%–30.91%) for S-IgG, and 51.63% (IQR, 31.25%–66.30%) for N-IgG (Figure 6, B–D).

## Discussion

The magnitude, functionality, and longevity of neutralizing antibody responses in patients with severe COVID-19 is poorly understood. In this study, we investigated the virus-neutralizing activities and antibody dynamic profiles of 104 patients with severe COVID-19 who were admitted to Wuhan Jinyintan Hospital during the first wave of the COVID-19 outbreak in Wuhan, China (32). Our findings provide evidence that the neutralizing antibodies to SARS-CoV-2 are persistent for at least 6 months in patients with severe COVID-19. Moreover, we identified that the level of RBD-IgG is capable of correlating with SARS-CoV-2–neutralizing activity in COVID-19 convalescent serum, consistent with previous studies (27, 33–35), which establishes the possibility that RBD-IgG be considered as the target for constantly monitoring vaccination effectiveness.

Elucidation of the antibody dynamic profiles in patients with COVID-19 not only reveals the prognosis of the disease, but also provides the experimental basis for practical applications of vaccines. Through the follow-up on early patients with severe COVID-19, we found that, although IgG levels of patients with severe COVID-19 at the convalescent phase were generally lower compared with those at the acute phase, the serum antibodies of more than 95% of patients were still able to neutralize SARS-CoV-2. Our findings are consistent with the previous observations that the neutralizing antibody titers in patients with COVID-19 decreased during the course of time after convalescence (8, 36). However, some reports showed that the neutralizing antibodies were consistent with the time frame in patients with COVID-19 (11, 37, 38). This discrepancy may be attributed to the different methods used for examining neutralizing activity by distinct research groups and the different sampling time-points during the acute phase and/or the convalescent phase, as well as to the fact that the cohorts were from different geographical regions. Overall, current studies, both those reported by others and ours, have shown that the serum collected from most of the patients with COVID-19 at the convalescent phase possess SARS-CoV-2–neutralizing activity, which supports the notion that the probability of reinfection with SARS-CoV-2 could be greatly reduced 6 months after disease onset and antibody acquisition (39, 40).

Interestingly, as the 104 patients in our study had been enrolled in the trial of lopinavir–ritonavir (31), with 50 in the lopinavir–ritonavir group and 54 in the standard-care group, AUC values were also used to compare the antibody levels in the 2 groups. Our results showed that no significant difference of IgG levels was found between these 2 groups at the convalescent phase (Supplemental Figure 2A). Moreover, the percentage of decrease in the RBD-IgG level from the acute to the convalescent phase in the lopinavir–ritonavir group was lower than that in the standard-care group (*P* = 0.0462, Supplemental Figure 2B), while the percentage of decreased S- and N-IgG levels between these 2 group showed no difference. Our results suggest that lopinavir–ritonavir may play a positive role in the production or maintenance of antibodies, which are consistent with previous studies that found the counts of B lymphocyte (CD19+) were higher in HIV patients who took lopinavir–ritonavir than in those who did not (41, 42).

It should be noted that our study has some limitations. First, the limited stock capacity of serum samples is an obstacle to us to further explore the correlation between RBD-IgG and virus-neutralizing activities in animal protection experiments. Second, more sampling points in a longer period after convalescence should be included for further assessment of RBD-, S-, and N-IgG levels and virus-neutralizing activities. In the future, we will keep following up on patients with COVID-19 at extended points in time to make more accurate and integrated judgments on the robustness and longevity of antibodies and the threshold for protection from reinfection.

In summary, our findings identified the magnitude, functionality, and longevity of antibody responses in the first wave of patients with COVID-19, which provide valuable data for the research community to better understand COVID-19-associated humoral immunity and would be beneficial to the efforts for developing vaccines.

## Methods

### Study design and participants.

All 104 subjects in our study had been enrolled in the randomized controlled clinical trial of lopinavir–ritonavir at Jinyintan hospital, Wuhan, China. The period of disease onset in patients was from December 20, 2019 to January 27, 2020 and the admission period was between January 17, 2020 and March 30, 2020. Diagnosis of SARS-CoV-2 infection was based on clinical diagnostic guidelines of the Chinese Health Commission (6th edition). Respiratory tract samples of the subjects were positive for nucleic acid of SARS-CoV-2, which were tested by real-time quantitative pCR (qRT-PCR) and viral pneumonia of each patient was confirmed with chest imaging by CT. The severity of the patients with COVID-19 was determined by the attending doctors based on the clinical diagnostic guidelines. In addition, demographic data of each subject were collected.

In our study, serum samples of patients with COVID-19 were collected during the acute and convalescent phases. The median period from disease onset to acute sampling point was 23 days (IQR, 20–27 days) and the median period from the acute sampling point to the convalescent sampling point was 172 days (IQR, 167–176 days). The collected serum samples were used for subsequent ELISA of RBD, S, and N-IgG and virus-neutralizing activities assay.

### Enzyme linked immunosorbent assay.

IgG antibodies against RBD, S, and N proteins were detected with anti-RBD, S, and N protein Human IgG ELISA Kit (AnyGo Technology Co., Ltd., XG100H8, XG100H7, and XG100H6) according to the manufacturer’s instructions. In short, serum samples of patients were diluted and added into RBD, S or N protein-coated plates, then incubated for 30 minutes. After washed with 1× PBST 4 times, horseradish peroxidase conjugated anti-human IgG antibodies were added and incubated for 15 minutes at room temperature. After additional rounds of washes, tetramethylbenzidine substrates were added and incubated for 5–10 minutes before termination. The plates were then read at 450 nm and 630 nm with F50 infiniteinfinite (TECAN).

### Microneutralization assay.

Vero E6 cells were seeded at 1 × 10^5^ per well in a 96-well culture plate at 37°C for 24 hours before use. Serial 2-fold dilutions of 50 μL of serum were prepared in a 96-well tissue culture plate in DMEM medium. An equal volume of SARS-CoV-2 working stock containing 200 TCID_50_ was added and the antibody-virus mixture was incubated at 37°C for 1 hour. Serum from healthy donors was used as negative controls. The antibody-virus mixture was then added into a 96-well microtiter plate containing equal volume of conﬂuent Vero E6 cells with 8 repeats and incubated at 37°C in CO_2_ incubator for 3 days. Cells infected with 100 TCID_50_ of SARS-CoV-2 and cells without infection were used as positive and uninfected controls, respectively. Cytopathic effect (CPE) in each well was observed daily and recorded on day 3 after infection. A virus back-titration was performed to assess the correct virus titer used in each experiment.

### Statistics.

All consecutive data are described as the medians (IQRs) or the means ± SD and categorical data are described as numbers (%). Unpaired 2-tailed Student’s t tests were used to compare 2 unpaired groups of variables. Paired 2-tailed Student’s t tests were used to compare the significance of paired samples. The 1-way ANOVA Student–Newman–Keuls multiple comparisons test was performed to test differences of continuous variables among multiple groups.

A *P* value less than 0.05 was considered significant (*: *P* < 0.05; **: *P* < 0.01; ***: *P* < 0.001; ****: *P* < 0.0001). The Pearson *correlation coefficient* (*r*) and the probability *P* value were calculated using GraphPad Prism, version 8.

### Study approval.

This study conformed to the 1975 Declaration of Helsinki guidelines and was approved by the Ethics Committees of Wuhan Jinyintan Hospital (KY-2020-83.01). Written informed consents were obtained from all involved patients.

## Author contributions

YW and XZ conceived the study. DZ, GW, YH, PL, and YQ designed the experiments. YH, PL, J. Zhou, YL, XH, QY, RH, XW, HS, PY, MY, and WJL performed the experiments. YH, PL,YQ, J. Zhang, and KP analyzed data and interpreted the results. The majority of the manuscript was written by YH, PL, and YQ, with some help from YW, XZ, DZ, and GW. All authors approved the final version of the manuscript. The order of the co–first authors was determined by their relative contribution to this study.

## Supplementary Material

Supplemental data

Supplemental Table 1

## Version 1 01/21/2021

In-Press Preview

## Version 2 02/22/2021

Electronic publication
