# Supplementary material for: Effective virus-neutralizing activities in antisera from the first wave of survivors of severe COVID-19
Source: JCI Insight. 2021 Feb 22;6(4):e146267. doi: 10.1172/jci.insight.146267 (PMC7934937; doi:10.1172/jci.insight.146267)
Supplement: Supplemental data [file jciinsight-6-146267-s232.pdf]

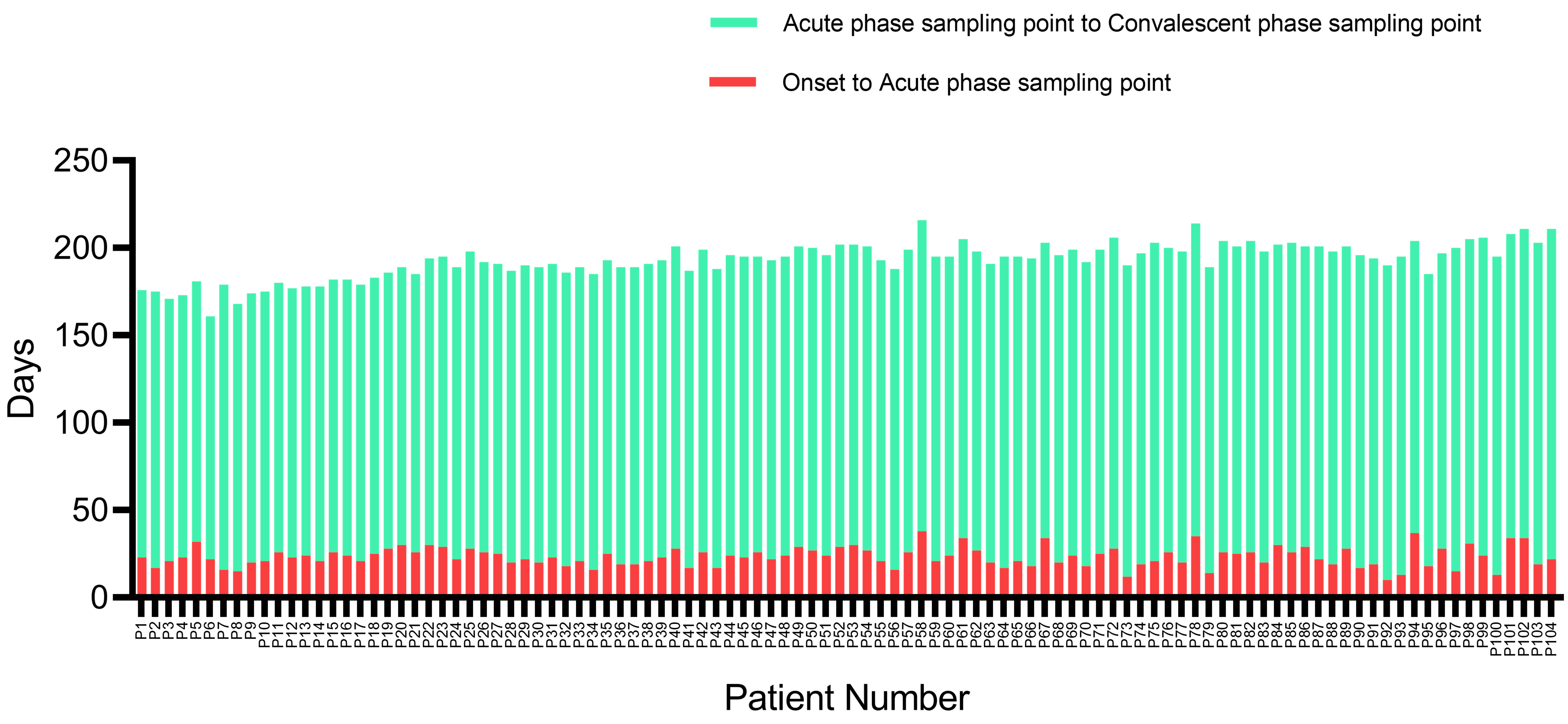
Acute phase sampling point to Convalescent phase sampling point
Onset to Acute phase sampling point
Days
250
200
150
100
50
0
Patient Number

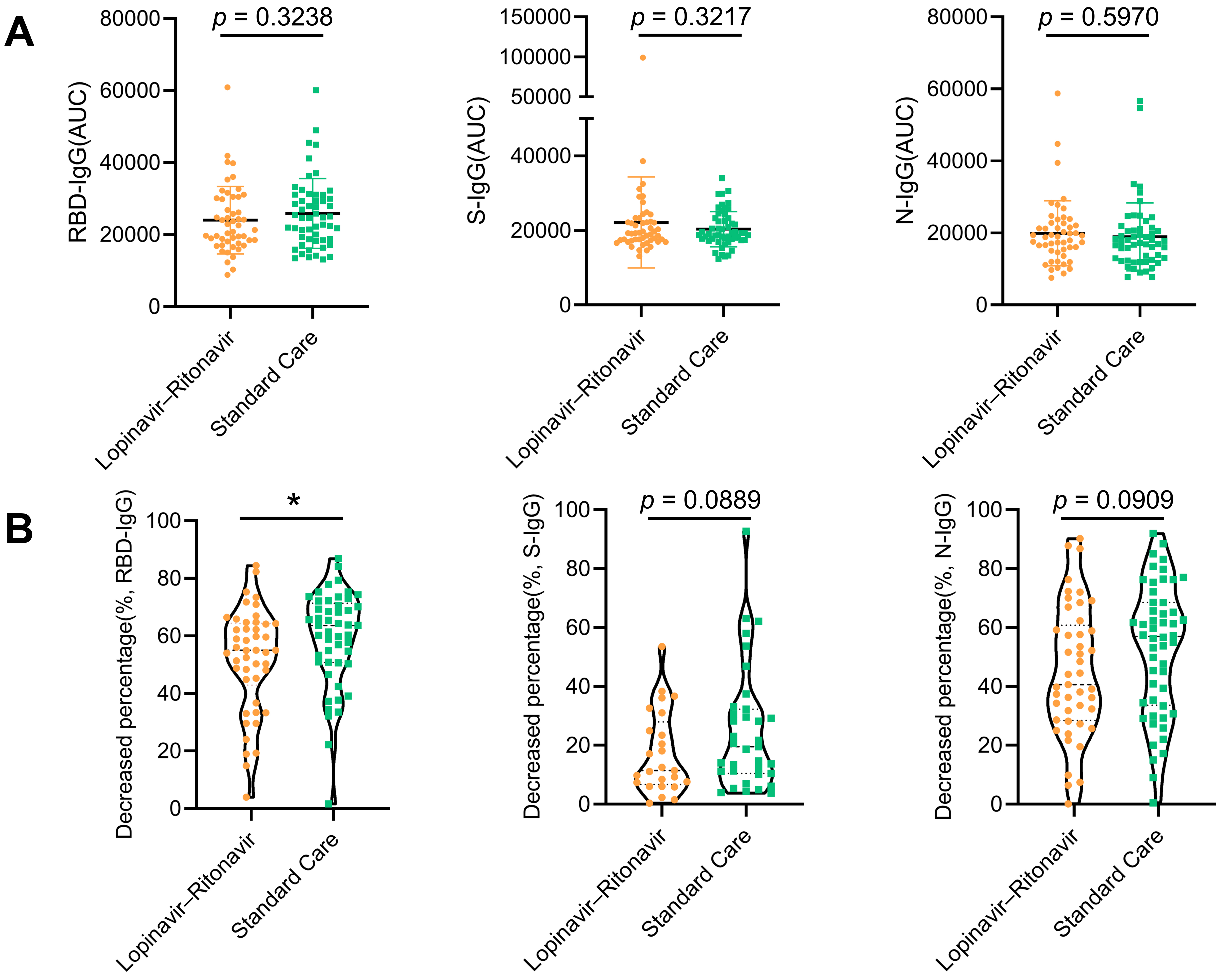
A
RBD-IgG(AUC)
p = 0.3238
S-IgG(AUC)
p = 0.3217
N-IgG(AUC)
p = 0.5970
Lopinavir–Ritonavir
Standard Care
B
Decreased percentage(%, RBD-IgG)
*
Decreased percentage(%, S-IgG)
p = 0.0889
Decreased percentage(%, N-IgG)
p = 0.0909

Supplemental Table S1. Clinical and Pathological Characteristics of Patients

**Age (years)**	56 (37-65)
**Sex (n, %)**	
Male	60 (71.4%)
Female	44 (57.7%)
**Inclusion Criteria**<br>(Fit any one of the two conditions)	1. oxygen saturation (Sao_2) ≤ 94% or less while breathing ambient air<br>2. ratio of the partial pressure of oxygen (Pao_2) to the fraction of inspired oxygen (Fio_2) of ≤ 300 mm Hg
**Oxygen Supplementation**	97 (93.3%)
**Ct Scan (Double lung damage) (n, %)**	104 (100%)
**Positive Nucleic Acid Tests (n, %)**	104 (100%)
**Course of Disease (Days)**	
Onset to discharge	25 (22, 30)
Onset to convalescent phase sampling point	195 (188, 201)
Onset to acute phase sampling point	23 (20, 27)
Acute phase sampling point to convalescent phase sampling point	172 (167, 176)
